# Hospital-Based Colorectal Cancer Survival Trend of Different Tumor Locations from 1960s to 2000s

**DOI:** 10.1371/journal.pone.0073528

**Published:** 2013-09-12

**Authors:** Yu-Jing Fang, Xiao-Jun Wu, Qian Zhao, Li-Ren Li, Zhen-Hai Lu, Pei-Rong Ding, Rong-Xin Zhang, Ling-Heng Kong, Fu-Long Wang, Jun-Zhong Lin, Gong Chen, Zhi-Zhong Pan, De-Sen Wan

**Affiliations:** 1 Department of Colorectal Surgery, Sun Yat-sen University Cancer Center, Guangzhou, China; 2 Department of Experimental Research, Sun Yat-sen University Cancer Center, Guangzhou, China; 3 State Key Laboratory of Oncology in South China, Sun Yat-sen University Cancer Center, Guangzhou, China; 4 Department of Statistics, School of Public Health, Guangzhou Medical University, Guangzhou, China; Baylor University Medical Center, United States of America

## Abstract

**Background:**

Our aim is to explore the trend of association between the survival rates of colorectal cancer (CRC) and the different clinical characteristics in patients registered from 1960s to 2000s. We hypothesized that the survival rate of CRC increases over time and varies according to anatomic subsites.

**Methods:**

Information from a total of 4558 stage T_(1-4)_N_(1-2)_M_0_ CRC patients registered from 1960s to 2008 were analyzed. The association of CRC overall survival with age, gender, tumor locations, time, histopathology types, pathology grades, no. of examined lymph nodes, the T stage, and the N stage was analyzed. The assessment of the influence of prognostic factors on patient survival was performed using Cox’s proportional hazard regression models.

**Results:**

From 1960 to 2008, the studied CRC patients included 2625 (57.6%) and 1933 (42.4%) males and females, respectively. These included 1896 (41.6%) colon cancers, and 2662 (58.4%) rectum cancers. The 5-year survival rate was 49%, 58%, 58%, 70%, and 77% for the time duration of 1960s, 1970s, 1980s, 1990s and 2000s, respectively. An increased 5-year survival rate was observed in the colon cancer and rectum cancer patients. Patients older than 60 years of age were more likely to develop colonic cancer (sigmoid) than rectum cancer (49.2% vs. 39.9%). The Cox regression model showed that only rectum cancer survival was related to time duration.

**Conclusion:**

The overall survival and 5-year survival rates showed an increase from the 1960s to 2000s. There is a trend of rightward shift of tumor location in CRC patients.

## Introduction

Cancers of the colon and rectum are the most common type of cancers and important contributors to cancer-related deaths in the Western world [1]. In 2008 389,700 males and 337,700 females in developed countries and 274,000 males and 232,400 females in developing countries were diagnosed with colorectal cancer (CRC), which is the third most commonly occurring cancer in males and the second most commonly occurring in females [2].

CRC is also one of the common malignant tumors occurring in China, with the CRC incidence and mortality rates ranking the second and the third, respectively, for females, and the third and the fourth, respectively, for males in China [3]. Over 172,000 new CRC cases and 99,000 deaths have been estimated to have occurred in 2005. CRC incidence was 15/10^5^ and 9.7/10^5^ for males and females, respectively, and CRC mortality was 8.6/10^5^ and 5.4/10^5^ for males and females, respectively [4]. CRC incidence and mortality have increased by 36.7% and 70.7%, respectively, from 1991 to 2005 in China [5]. The incidence rank is the same worldwide; however, CRC mortality has declined in the developed countries because the incidence has not increased and also because the survival has improved [2]. Moore et al. reported data on cancer incidences in five continents in 1982 and 2002, which showed that China, including Shanghai, and Hong Kong, have higher rates for rectal cancer compared to that in the US [6]. Takada et al. analyzed the time trend of CRC in Japan between 1974 and 1994 according to the site of the tumor within the colon or the rectum, and found that the percentage of occurrence of right-sided colon cancer in colon cancer cases was stable in men, but showed an increasing trend in women. Li et al. analyzed the Chinese data between 1980 to 1999 and found that a proximal shift due to the increasing proportion of ascending and transverse colon occurred in CRC patients [7].

We undertook this study on patients with CRC at stage T_(1-4_)N_(1-2_)M_0_ to examine the trend in survival rates of colon and rectum cancer in relation to the different clinical characteristics. It was hypothesized that the survival rate of CRC increases over time and varies according to anatomic subsites (the colon and rectum).

## Methods

### Study patients

The hospital based Chinese CRC database was created by the Colorectal Cancer Registry Center in Southern China, Sun Yat-sen University Cancer Center. The study was performed following approval by the ethic committee of Sun Yat-sen University Cancer Center. We were informed that it is not necessary to get signatures of patients’ on the informed consent form, since according to the current Chinese medical regulations, the process of the whole study is non-invasive and without any effect on the patients’ benefit. The database comprises the in-patients’ clinicopathological information and follow-up information, which included information of over 8000 CRC patients registered from the 1960s to 2011. In this study, we used 4558 CRC cases from the database. Patients with stage T_(1-4_)N_(1-2_)M_0_ tumors (as defined by the International Union Against Cancer, tumor-node-metastasis, TNM staging criteria, version 6, 2002) [8] who had undergone curative surgical resection were included in this study. Cases with known hereditary non-polyposis CRC, familial adenomatous polyposis, and previous history of malignancy were excluded. The information was further categorized into colon cancer (ascending colon cancer, transverse colon cancer, descending colon cancer, sigmoid colon cancer) and rectum cancer.

### Follow-up

The patients were followed up every 3 months for the first 2 years and then every 6 months for the next 3 years and finally annually. The median duration of follow-up for the entire population was 61 months (range: 1–660 months).

### Statistical analysis

We analyzed the trends in age, gender, tumor locations, time, survival, and so on. We investigated the association of CRC survival with age, gender, tumor locations, time, histopathology types, pathology grades, the no. of examined lymph nodes, the T stage, and the N stage.

Overall survival (OS) and 5-year survival were the end-points of this study. The Kaplan-Meier estimate and log-rank tests were used to describe and compare OS based on lifetime data. The assessment of the influence of prognostic factors on patient survival was performed using Cox’s proportional hazard regression models. *P* ≤ 0.05 indicated statistically significant differences.

## Results

From 1960 to 2008, 4558 CRC patients had been registered in the database, of which 2625 (57.6%) and 1933 (42.4%) were males and females, respectively. The age ranged from 7 to 95 years old, with the median age being 56 years old. A total of 1445 (31.7%), 1227 (26.9%), 1149 (25.2%), 654 (14.4%), and 80 (1.8%) patients belonged to the age groups of less than 50 years old, 50–59 years old, 60–69 years old, 70–79 years old, and older than 80 years old groups, respectively. The CRC cases included 1896 (41.6%) colon cancers cases and 2662 (58.4%) rectum cancers cases.

### Characteristic trends of the study population diagnosed from the 1960s to 2008

The characteristics of the study patients in each decade are shown in Table 1. The proportion of patients diagnosed before the age of 50 years dropped from 1960 to 2008 (54.1% to 27.3%). The proportion of patients diagnosed over the age 60 years increased over time. The proportion of gender did not change significantly over time. The proportion of patients with sigmoid colon cancer showed a significant increase in the 2000s (20.7%) compared with that in the other decades (13.8%) (p = 0.001). The rectum cancer significantly decreased in the 2000s (54.4%) corresponding to that in the other decades (63.6%) (p = 0.001).

**Table 1:** Characteristics of the study population diagnosed from the 1960s to 2008.

	**Total**	**60s**	**70s**	**80s**	**90s**	**00s**	**P**
Total	4558	98/2.15	294/6.45	462/10.1	1124/24.7	2580/56.6	
Age							0.0001
<50	1445/31.7	53/54.1	158/54.3	178/38.5	352/31.3	704/27.3	
50-59	1227/26.9	31/31.6	82/28.2	155/33.5	288/25.6	671/26.0	
60-69	1149/25.2	13/13.3	49/16.8	90/19.5	321/28.6	676/26.2	
70-79	654/14.4	1/1.0	2/0.7	34/7.4	141/12.5	476/18.4	
>80	80/1.8	0	0	5/1.1	22/2.0	53/2.1	
Gender							0.807
Male	2625/57.6	59/60.2	167/56.8	260/56.3	636/56.6	1503/58.3	
Female	1933/42.4	39/39.8	127/43.2	202/43.7	488/43.4	1077/41.7	
Tumor site							0.0001
Ascending	682/15	15/15.3	47/16.0	69/14.9	150/13.3	401/15.5	
Transverse	157/3.4	7/7.1	14/4.8	13/2.8	30/2.7	93/3.6	
Descending	249/5.5	6/6.1	13/4.4	23/5.0	60/5.3	147/5.7	
Sigmoid	808/17.7	13/13.3	36/12.2	54/11.7	170/15.1	535/20.7	
Rectum	2662/58.4	57/58.2	184/62.6	303/65.6	714/63.5	1404/54.4	
T stage							0.0001
1	240/5.3	1/1.1	7/2.4	18/3.9	45/4.0	169/6.6	
2	1126/24.9	32/34	87/30.2	170/37.1	342/30.6	495/19.4	
3	1860/41.2	50/53.2	186/64.6	241/52.6	554/49.6	829/32.4	
4	1289/28.5	11/11.7	8/2.8	29/6.3	177/15.8	1064/41.6	
N stage							0.0001
0	3012/68.6	70/76.9	214/82.9	348/81.5	761/73.0	1619/62.9	
1	938/21.4	20/22	28/10.9	50/11.7	202/19.4	638/24.8	
2	443/10.1	1/1.1	16/6.2	29/6.8	79/7.6	318/12.3	

For the patients diagnosed using TNM, who have been compared for the proportion of the diagnosis with the T stage and the N stage, we found that early diagnosis with the T1 stage and the T4 stage increased significantly in the 2000s, and that with the T2 stage and the T3 stage decreased significantly in 2000s. The early diagnosis with the N1 and N2 stage also increased significantly in the 2000s.

**Figure 1:**
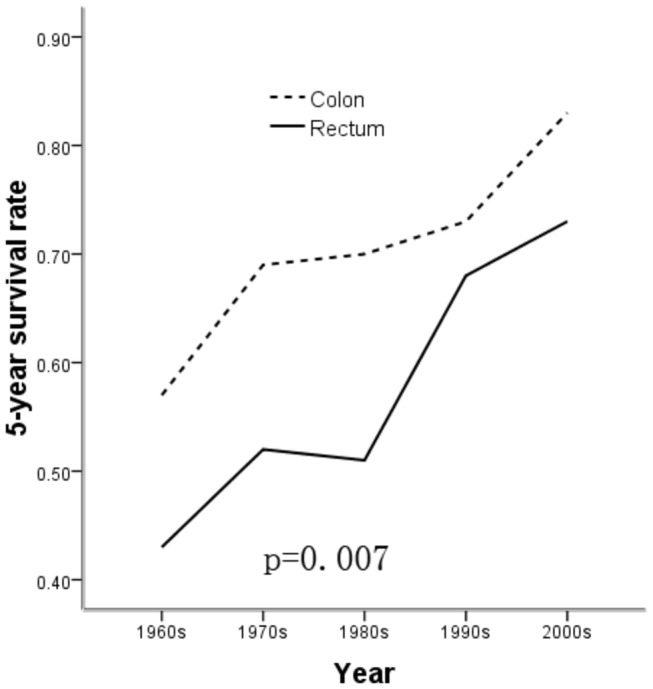
The 5-year survival rate was compared between tumors at the colon site and the rectum site (p=0.007).

The 5-year survival rates were 49%, 58%, 58%, 70%, and 77% for the time duration of 1960s, 1970s, 1980s, 1990s and 2000s, respectively, which showed that the 5-year survival rates increased after 2000. Figure 1 shows that the 5-year survival rate increased in both the patients with colon cancer and rectum cancer, but was significantly higher in the colon cancer patients (p = 0.007).

### Characteristics trend of the study population diagnosed with tumor in different locations

The characteristics of the study patients diagnosed with tumors in different locations are shown in Table 2. The proportion of age over 60 years old (including group 60–69, 70–79, and >80 years old) significantly increased in sigmoid colon cancer patients (30.3%, 16.5% and 2.4%, respectively) than in rectum cancer patients (24.6%, 13.8% and 1.5%, respectively) (49.2% vs. 39.9%, p = 0.001). The proportion of age younger than 59 years old decreased in descending and sigmoid colon cancer patients. The proportion of the T1 and T2 stage increased in the sigmoid colon cancer and rectum cancer patients, and that of the T3 stage and T4 stage increased in the ascending and transverse colon cancer patients, respectively. However, the T4 stage decreased in the sigmoid colon cancer and rectum cancer patients. We have also compared the 5-year survival rates between each tumor location. Table 2 showed that the 5-year survival rate increased in the ascending, descending, sigmoid colon cancer and rectum cancer patients, however, not in the transverse colon cancer patients from the 1960s to 2000s.

**Table 2:** Characteristics of the study populations with respect to different tumor sites.

	Total	Ascending	Transverse	Descending	Sigmoid	Rectum	P
Total	4558	682/15	157/. 4	249/5.5	808/17.7	2662/58.4	
Age							0.001
<50	1445/31.7	237/34.8	52/33.1	90/36.1	192/23.8	874/32.9	
50-59	1227/26.9	178/26.1	47/29.9	60/24.1	219/27.1	723/27.2	
60-69	1149/25.2	153/22.4	34/21.7	62/24.9	245/30.3	655/24.6	
70-79	654/14.4	100/14.7	22/14	33/13.3	133/16.5	366/13.8	
>80	80/1.8	14/2.1	2/1.3	4/1.6	19/2.4	41/1.5	
Gender							0.805
Male	2625/57.6	404/59.2	93/59.2	147/59	457/56.5	1524/57.3	
Female	1933/42.4	278/40.8	64/40.8	102/41	351/43.4	1138/42.7	
Outcome							0.0001
Survival	3014/66.6	474/69.9	107/68.6	179/72.2	604/75.4	1650/62.4	
Death	1514/33.4	204/30.1	49/31.4	69/27.8	197/24.6	995/37.6	
T stage							0.0001
1	240/5.3	12/1.8	5/3.2	9/3.6	43/5.4	171/6.5	
2	1126/24.9	97/14.4	25/16.1	37/15	161/20.1	806/30.6	
3	1860/41.2	302/44.8	59/38.1	107/43.3	330/41.2	1062/40.3	
4	1289/28.5	263/39	66/42.6	94/38.1	267/33.2	599/22.7	
N stage							0.030
0	3012/68.6	468/70.9	112/72.7	184/77	544/69.2	1704/66.7	
1	938/21.4	131/19.8	27/17.5	41/17.2	170/21.6	569/22.3	
2	443/10.1	61/9.2	15/9.7	14/5.9	72/9.2	281/11	
5-year survival rate		77%	79%	79%	78%	67%	
60s	49%	54%	83%	67%	42%	43%	
70s	58%	77%	77%	54%	63%	52%	
80s	58%	62%	83%	77%	72%	51%	
90s	70%	74%	68%	79%	72%	68%	
00s	77%	82%	82%	84%	84%	73%	

### Effect of each factor in different tumor locations on overall survival

**Figure 2:**
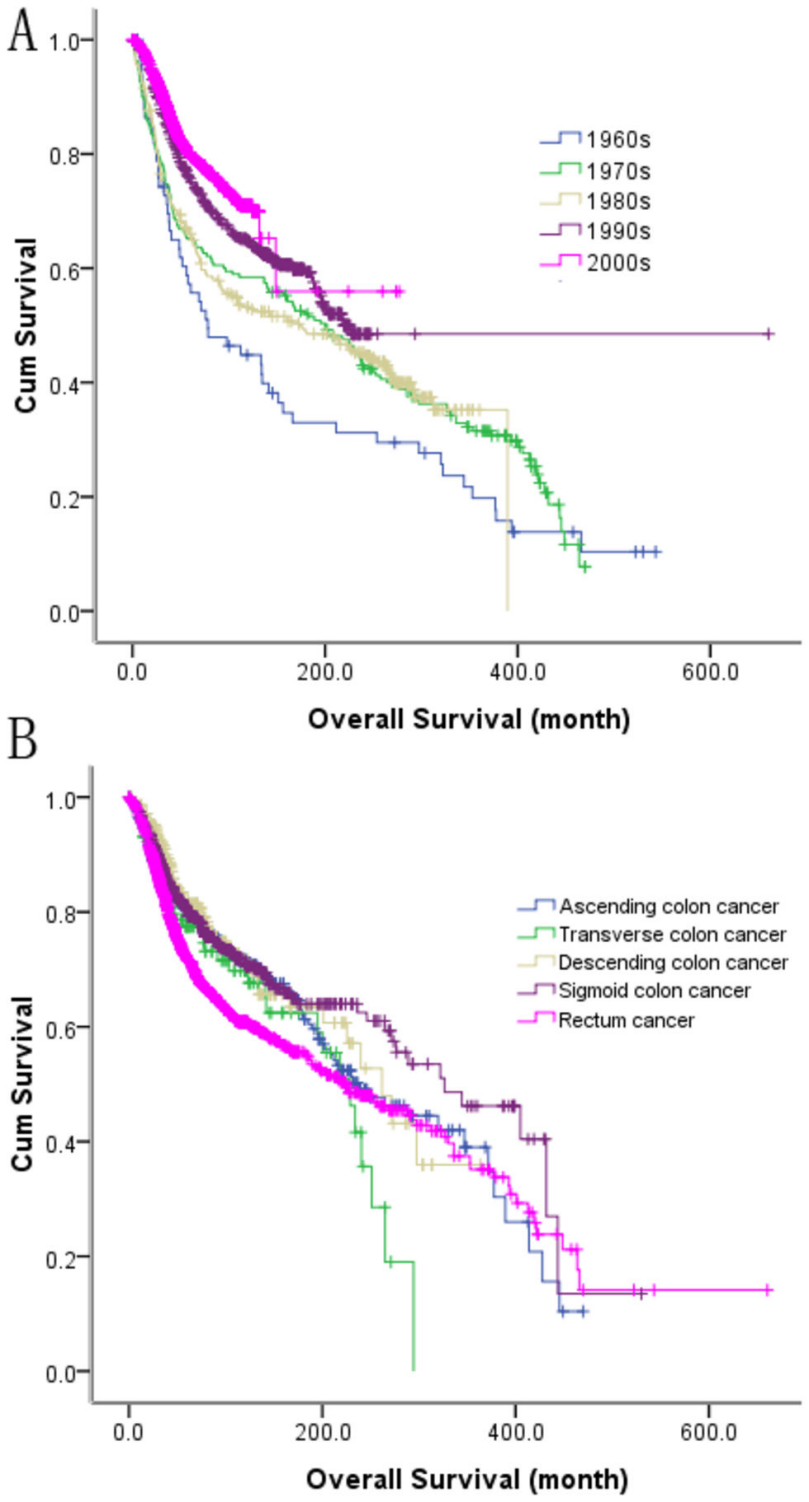
The survival curves for patients in different time durations (A) and with different tumor locations (B).

The multivariate Cox models for OS included the following variables: age (uni-, *p* = 0.0001), gender (uni-, *p* = 0.001), tumor locations (uni-, *p* = 0.0001), tumor size (uni-, *p* = 0.007), histopathology types (uni-, *p* = 0.0001), pathology grades (uni-, *p* = 0.0001), the T stage (uni-, *p* = 0.0001), the N stage (uni-, *p* = 0.0001), LN No (uni-, *p* = 0.0001). Table 3 shows the final results of multivariate Cox analysis for age (p = 0.0001), gender (p = 0.006), time duration (p = 0.0001) (Figure 2), tumor locations (p = 0.0001) (Figure 2), pathology grades (p = 0.001), no. of examined lymph nodes (p = 0.0001), the T stage (p = 0.0001), and the N stage (p = 0.0001).

**Table 3:** Multivariate Cox regression analysis for overall survival in patients with different tumor locations.

Terms	Hazard ratio	95%CI	P
Age			0.0001
<50	1		
50-59	1.036	0.851-1.261	0.724
60-69	1.507	1.249-1.819	0.0001
70-79	1.941	1.577-2.389	0.0001
>80	2.616	1.717-3.985	0.0001
Gender			
Male	1.215	1.056-1.398	0.006
Female	1		
Time duration			0.0001
1970s	1		
1980s	2.113	1.042-4.284	0.038
1990s	1.438	0.716-2.886	0.307
2000s	1.099	0.537-2.250	0.797
Tumor locations			0.0001
Ascending	0.668	0.529-0.844	0.001
Transverse	0.799	0.518-1.231	0.308
Descending	0.611	0.420-0.888	0.01
Sigmoid	0.725	0.589-0.891	0.002
Rectum	1		
Pathology grade			0.001
Grade I	1		
Grade II	1.050	0.831-1.327	0.682
Grade III	1.642	1.245-2.166	0.0001
Examined lymph nodes			0.0001
N=0	1.667	1.274-2.182	0.001
N<12	1.090	0.906-1.310	0.361
N≥12	1		
T stage			0.0001
1	1		
2	1.368	0.845-2.214	0.202
3	1.935	1.205-3.107	0.006
4	2.267	1.403-3.662	0.001
N stage			0.0001
0	1		
1	2.349	1.955-2.821	0.0001
2	3.391	2.727-4.216	0.0001

According to the above results, the tumor location is a risk factor for OS. We have conducted another multivariate Cox regression analysis which is based on the different tumor locations. The results of the multivariate Cox regression analysis are shown in Table 4. In the ascending colon cancer, the results showed that only the N stage (N1, HR = 2.93, 95% CI 1.728–4.969, p = 0.0001; N2, HR = 2.264, 95% CI 1.124–4.56, p = 0.022) was an independent risk factor for OS. In the transverse colon cancer, the gender (males, HR = 4.473, 95% CI 1.244–14.077, p = 0.022) and the pathology grade (Grade III, HR = 13.18, 95% CI 1.474–117.9, p = 0.021) were the independent risk factors for OS. In the descending colon cancer, only the pathology grade (p = 0.012) was an independent risk factor for OS. The age (>80 years, HR = 2.85, 95% CI 1.126–7.212, p = 0.027), examined lymph nodes (N = 0, HR = 2.039, 95% CI 1.004–1.144, p = 0.049), and the N stage (N1, HR = 2.2, 95% CI 1.41–3.45, p = 0.001; N2, HR = 3.81, 95% CI 2.16–6.71, p = 0.0001) were significantly related to OS in the sigmoid colon cancer. The age (60–69 years, HR = 1.43, 95% CI 1.103–1.8, p = 0.006; 70–79 years, HR = 2.22, 95% CI 1.72–2.89, p = 0.0001; >80 years, HR = 3.104, 95% CI 1.79–2.359, p = 0.0001), gender (males, HR = 1.243, 95% CI 1.04–1.484, p = 0.016), time duration (1980s, HR = 3.319, 95% CI 1.157–9.524, p = 0.026), pathology grades (Grade III, HR = 2.024, 95% CI 1.406–2.915, p = 0.0001), the T stage (T = 3, HR = 1.876, 95% CI 1.07–3.288, p = 0.028; T = 4, HR = 2.192, 95% CI 1.234–3.891, p = 0.007), the N stage (N1, HR = 2.382, 95% CI 1.881–3.018, p = 0.0001; N2, HR = 3.602, 95% CI 2.744–4.729, p = 0.0001), and the examined lymph nodes (N = 0, HR = 1.733, 95% CI 1.232–2.437, p=0.002) were independent risk factors for OS in the rectum cancer.

**Table 4:** Hazard ratios (HR) and 95% confidence intervals (CIs) for each tumor site patient estimated using multivariate analysis of Cox regression model.

	Ascending	Transverse	Descending	Sigmoid	Rectum
	P/HR/95%CI	P/HR/95%CI	P/HR/95%CI	P/HR/95%CI	P/HR/95%CI
Age				**0.038**	**0.0001**
<50				/1	/1
50-59				0.49/0.823/	0.713/1.047/
				0.473-1.432	0.82-1.34
60-69				0.142/1.459/	**0.006/1.43/**
				0.881-2.418	**1.103-1.8**
70-79				0.371/1.313/	**0.0001/2.22/**
				0.722-2.388	**1.72-2.89**
>80				**0.027/2.85/**	**0.0001/3.104/**
				**1.126-7.212**	**1.79-2.359**
Gender					
Male		**0.022/4.473/**			**0.016/1.243/**
		**1.244-16.077**			**1.04-1.484**
Female		/1			/1
Years					**0.0001**
70s					/1
80s					**0.026/3.319/**
					**1.157-9.524**
90s					0.34/1.656/
					0.587-4.671
2000-08					0.567/1.362/
					0.473-3.923
Pathology grade		**0.013**	**0.012**		**0.0001**
Grade I		/1	/1		/1
Grade II		0.612/1.583/	0.383/0.495/		0.178/1.236/
		0.268-9.349	0.102-2.405		0.908-1.682
Grade III		**0.021/13.18/**	0.487/1.853/		**0.0001/2.024/**
		**1.474-117.9**	0.325-10.565		**1.406-2.915**
Examined lymph				**0.0001**	**0.005**
Nodes					
N=0				**0.049/2.039/**	**0.002/1.733/**
				**1.004-1.144**	**1.232-2.437**
N< 12				0.763/0.937/	0.223/1.152/
				0.566-1.518	0.918-1.446
N ≥ 12				/1	/1
T stage					**0.0001**
1					/1
2					0.301/1.348/
					0.765-2.375
3					**0.028/1.876/**
					**1.07-3.288**
4					**0.007/2.192/**
					**1.234-3.891**
N stage	**0.0001**			**0.0001**	**0.0001**
0	/1			/1	/1
1	**0.0001/2.93/**			**0.001/2.2/**	**0.0001/2.382/**
	**1.728-4.969**			**1.41-3.45**	**1.881-3.018**
2	**0.022/2.264/**			**0.0001/3.81/**	**0.0001/3.602/**
	**1.124-4.56**			**2.16-6.71**	**2.744-4.729**

## Discussion

In our study, the patients have dramatically increased from the 1960s to 2000s in the Sun Yat-sen University cancer center. From Table 1, we found that patients with stage I-II (N = 0) cancers decreased dramatically, at the same time, the state III CRC patients increased obviously from 1960s to 2000s. It is known that some of the developed and westernized Asian countries have already experienced a rapidly rising trend in CRC. In Japan, the incidence has also increased since the 1960s, and similar patterns have been reported in Hong Kong, Taiwan [9] and Singapore [2,10,11]. It is very difficult to obtain epidemiologic data for China. A study in urban Shanghai [12] reported that colonic and rectal cancers together rank as the third commonest malignant disorder in the city. The rising incidence of CRC in Henan province has also been recognized [13]. The reason is not only the environmental and lifestyle changes in China, but also the increased diagnostic levels and standardization of cancer treatment (pharmacological interventions and surgical therapies).

Our data showed a trend of rightward shift of tumor location in CRC patients after 2000. Table 1 shows that only the proportion of sigmoid colon cancer significantly increased in 2000 (20.7%) compared with the other decades (13.8%, p = 0.001), and the rectum cancer significantly decreased in 2000s (54.4%) corresponding to that in the other decades (63.6%, p = 0.001). No significant change was observed for proximal cancers in patients over time. Data also showed that patients older than 60 years were more likely to develop colonic cancer than rectum cancer. This result is similar to that observed in Japanese patients. Takada et al. also showed that the percentage of patients over the age of 70 years showed an increased rightward shift in the tumor location [14]. In the last part of the results, we conducted the Cox regression analysis for the patients with different tumor locations. It showed that only the rectum cancer was related to difference in time duration. In a retrospective cohort study, 690 U.S.A. patients and 870 Chinese patients were compared, which showed that proximal cancers were more common in the US than in the Chinese patients [15]. According to the Japanese Society and the Seoul Cancer Registry, increased proportion of proximal colon cancers are observed in Japanese in both sexes at all ages [14] and in Korean women older than 60 years [16]. The data from Hong Kong also showed that 5.1% of the asymptomatic population had advanced neoplastic lesions in the proximal colon [17]. However, the distribution shift of CRC has not been found in preliminary data from the Singapore Cancer Registry, which analyzed the data between 1968 and 1992 [18]. It is very difficult to explain the reason for the proximal shift of CRC. Although the reason for the tumor site shift is still unclear, many experts have suggested that the higher incidence of colon cancer might be dietary habits changes, etiological changes, and the economic growth, followed by wider availability of colonoscopy and ageing populations in many Asian countries [19-23].

In conclusion, based on the results from our study, over the 50-year period, the 5-year survival of CRC patients (49%, 58%, 58%, 70%, and 77%, from the 1960s to 2000s, respectively) has improved between the 1960s and 2000s. It might be due to the advances in surgical techniques, chemotherapy, target treatment, life style, and physical activity [24-29]. The 5-year survival was 77%, 79%, 79%, and 78% for ascending, transverse, descending, and sigmoid, respectively. However, for rectum cancer, 5-year survival was 67%. Among the overall data, a better 5-year survival was statistically significantly associated with tumor location in the colon than in the rectum (p = 0.007). However, in our study, we did not find significantly better 5-year survival of sigmoid than the other location of colon, similar to the results of O’connell [30,31].

There is still a lack of thorough epidemiology data in China. As we know that cancer screening is highly important to decrease the morbidity and mortality of CRC [32-34]. In recent years, the Chinese government has attached great importance to cancer screening. The hospital-based registries of CRC have already been established by the Ministry of Public Health. Health education on cancer prevention, establishment of a good lifestyle and behavior, appropriate and balanced diet, active treatment of pre-carcinogenic lesion, and periodic routine follow-ups are very important terms for people to know and to follow from now on. The method of screening not only includes fecal occult blood test (FOBT) [35,36], but a fecal immunochemical test (FIT) has also been demonstrated to have higher sensitivity [37-39]. Further improvement is expected by testing for molecules in stool or blood that are more directly related to the cancer process.
